# Idiosyncratic and dose-dependent epistasis drives variation in tomato fruit size

**DOI:** 10.1126/science.adi5222

**Published:** 2023-10-19

**Authors:** Lyndsey Aguirre, Anat Hendelman, Samuel F. Hutton, David M. McCandlish, Zachary B. Lippman

**Affiliations:** 1 Cold Spring Harbor Laboratory, School of Biological Sciences, Cold Spring Harbor, NY, USA.; 2 Cold Spring Harbor Laboratory; Cold Spring Harbor, NY, USA.; 3 Gulf Coast Research and Education Center, University of Florida, Wimauma, FL, USA.; 4 Howard Hughes Medical Institute, Cold Spring Harbor Laboratory, Cold Spring Harbor, NY, USA.

## Abstract

Epistasis between genes is traditionally studied using mutations that eliminate protein activity, but most natural genetic variation is in cis-regulatory DNA and influences gene expression and function quantitatively. Here, we use natural and engineered cis-regulatory alleles in a plant stem cell circuit to systematically evaluate epistatic relationships controlling tomato fruit size. Combining a promoter allelic series with two other loci, we collected over 30,000 phenotypic data points from 46 genotypes to quantify how allele strength transforms epistasis. We revealed a saturating dose-dependent relationship, but also allele-specific idiosyncratic interactions, including between alleles driving a step change in fruit size during domestication. Our approach and findings expose an underexplored dimension of epistasis, where cis-regulatory allelic diversity within gene regulatory networks elicits non-linear, unpredictable interactions that shape phenotypes.

## One-Sentence Summary

Epistasis analysis across a cis-regulatory allelic series reveals unpredictable, non-linear interactions that shape trait variation.

Epistasis analysis is an essential tool for discovering functional relationships between genes. At its simplest, an epistatic interaction is determined by testing if the phenotypic effect from one gene mutation modifies (e.g. suppresses or enhances) the phenotypic effect of another (1, 2). Historically, epistasis studies have relied on mutations with strong effects on protein function and phenotype, typically obtained from natural mutants or laboratory mutagenesis experiments (1–4). Recently, high-throughput engineering and the combination of gene deletions in yeast have allowed for the characterization of global interaction networks (5–10). While these and related studies, including those now leveraging genome-editing technologies in more complex systems (11–15), can dissect epistasis at scale, they do not address how cis-regulatory mutations, which are pervasive in genomes and responsible for the majority of functional variation in organisms (16–19), impact epistatic relationships and the phenotypes they control.

Compared to protein-coding mutations, cis-regulatory mutations more often produce graduated effects on gene function that alter expression level or timing (16, 20, 21). Across species, natural variation in gene expression is predominantly associated with regulatory sequences of the differentially expressed genes (16, 19, 22), and cis-regulatory variants are the primary contributors to phenotypic diversity (16, 18). Despite their critical functional role, few studies have explored epistatic relationships in the context of cis-regulatory variation (5, 10, 23), and none have done so in depth. Due to limited allelic variation at known interacting genes and inadequate quantitative phenotyping power in most model systems, we lack an understanding of how this widespread genetic variation affects the form and magnitude of epistasis.

We addressed this knowledge gap by taking advantage of the *CLAVATA-WUSCHEL* (*CLV-WUS*) gene regulatory circuit in plants (24). *CLV-WUS* controls stem cell proliferation in small groups of cells at shoot apices called meristems, which enable the continuous development of new tissues and organs during post-embryonic growth (24). Using tomato as a model, we asked how previously documented epistatic interactions in this circuit are affected by replacing one critical gene, *CLAVATA3* (*CLV3*), with a wide range of stronger and weaker cis-regulatory alleles.

*CLV3* encodes a small signaling peptide that restricts stem cell proliferation and meristem size by repressing *WUS*, a stem-cell-promoting homeobox transcription factor gene (24). In a negative feedback loop, *WUS* suppresses its own expression by activating *CLV3* to restrict stem cell proliferation and maintain meristem size throughout development (24). Epistasis between *CLV3* and *WUS* was first established using mutants in the model *Arabidopsis thaliana* (25), and our previous CRISPR-Cas9 mutagenesis of the tomato orthologues has shown this relationship is conserved (26–28). In both systems, meristem growth in *wus* mutants ceases during vegetative development, resulting in a failure to develop flowers and fruits. Conversely, meristems of *clv3* mutants become greatly enlarged, leading to more flowers, fruits, and their associated organs, including seed compartments known as locules. In a classical suppression epistatic relationship, *wus* mutations completely mask *clv3* phenotypes (i.e. *clv3 wus* double mutants are indistinguishable from *wus* single mutants). Tomato also features an additional layer of epistasis involving a paralog of *SlCLV3* (*Solanum lycopersicum*, denoted by ‘*Sl*’) in the *CLV3/EMBRYO-SURROUNDING REGION* (*CLE*) gene family, *SlCLE9* (27). *SlCLE9* is an ancient paralog, whose natural allelic state in wild and domesticated tomatoes is a partial loss-of-function (i.e. hypomorphic) due to changes in both its protein sequence and cis-regulatory control (27, 29). While null mutants of *Slcle9* are indistinguishable from wild-type plants, *Slclv3* is strongly enhanced by *Slcle9*, demonstrating a canonical unequal redundancy (30) epistatic relationship between these paralogs.

**Fig. 1.**
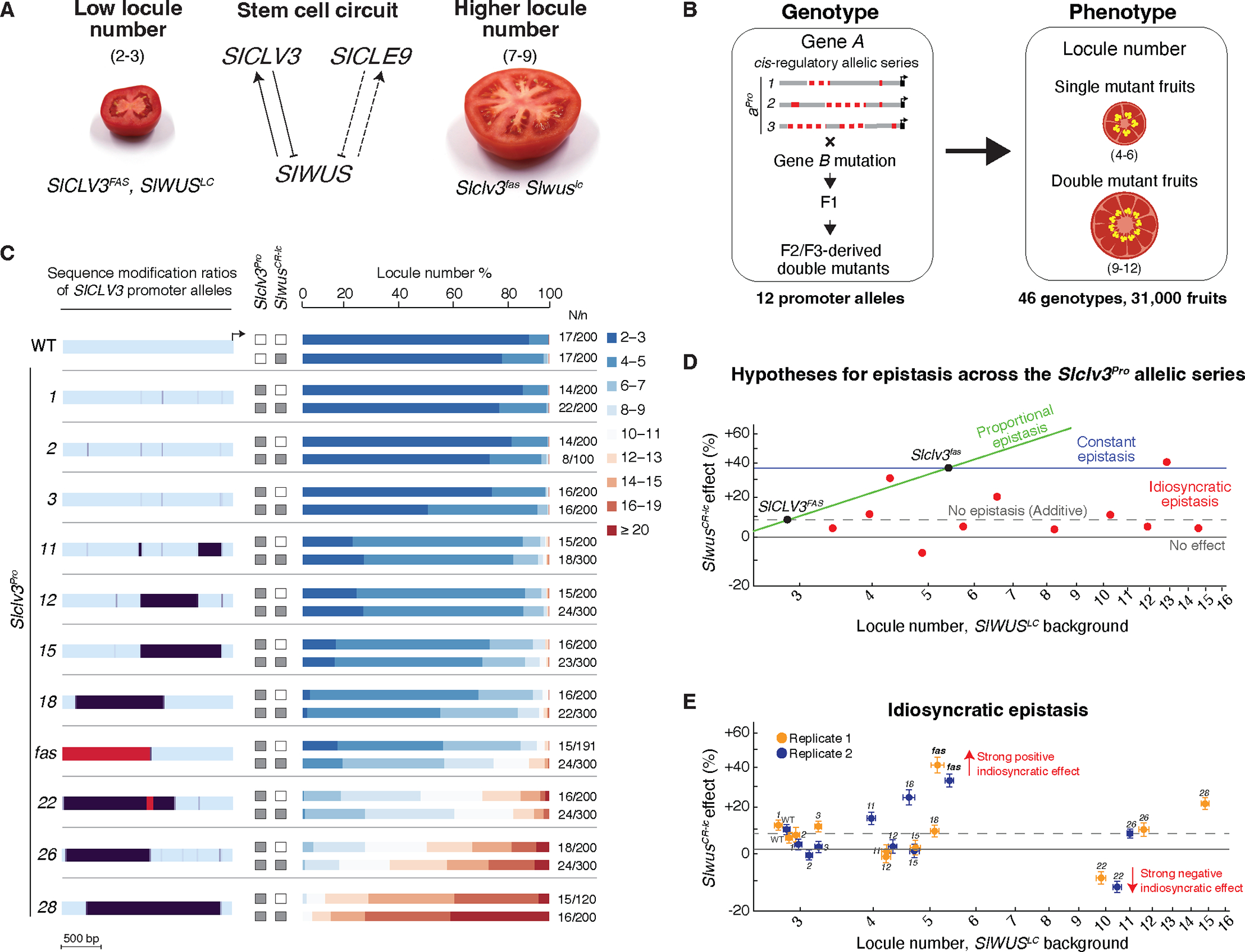
A promoter allelic series of the fruit size gene *SlCLV3* reveals idiosyncratic epistasis. (**A**) The *SlWUS-SlCLV3* circuit and the paralog *SlCLE9* control locule number. Fruits of wild type (WT, left) and *Slclv3*^*fas*^ *Slwus*^*lc*^ double mutants (right). Dashed lines and numbers indicate locules. (**B**) Experimental design. (**C**) Heatmap of *SlCLV3* promoter region encompassing 11 *Slclv3* promoter (*Slclv3*^*Pro*^) alleles. Purple intensity in 20 bp windows indicates ratios of sequence change relative to WT (cyan). Red: inversion. Stacked bar charts are percentage of fruits having each locule number range. White/Gray boxes indicate WT and mutant genotype for each gene, respectively. Replicated plants/fruits (N/*n*). (**D**) Epistasis models between *Slwus*^*CR-lc*^ and the *Slclv3*^*Pro*^ alleles, depicted by plotting percent change of double mutants against mean log locule numbers of *Slclv3*^*Pro*^ mutants. Combined effect of *Slwus*^*lc*^ and *Slclv3*^*fas*^ is indicated. (**E**) *Slwus*^*CR-lc*^ effect on mean log locule number (*Slwus*^*CR-lc*^ *Slclv3*^*Pro*^ genotypes compared to *SlWUS*^*LC*^ *Slclv3*^*Pro*^ genotypes), plotted against mean log locule number of the corresponding *SlWUS*^*LC*^ *Slclv3*^*Pro*^ genetic background (error bars indicate ±1 standard error). Data are from two replicated trials, except for *Slclv3*^*Pro-28*^ (see also fig. S2A, and tables S2 and S3). Red arrows show strongest idiosyncratic effects, including positive synergism between *Slclv3*^*fas*^ and *Slwus*^*lc*^.

Although conventional protein-coding null mutations were used to characterize these epistatic relationships, two natural cis-regulatory alleles of *SlWUS* and *SlCLV3,* are also known to exhibit a strong epistatic interaction (26). In fact, this interaction played an important role in the expansion of fruit size via an increase in locule number that occurred during tomato domestication (26, 31). Specifically, the ancestral state of tomato, which is maintained in many cultivated genotypes, is to produce fruits with two or three locules (Fig. 1A). A quantitative trait locus (QTL) allele known as *locule number* (*lc*) then emerged in the progenitor of modern tomatoes (31). This allele disrupts a repressor element downstream of *SlWUS* (*Slwus*^*lc*^), leading to a weak gain-of-function and a slight increase of approximately 10% in the number of three-locule fruits (26). Subsequently, another QTL allele, *fasciated* (*fas*), arose in the form of an inversion that reduces the activity of the *SlCLV3* promoter (*Slclv3*^*fas*^) (26, 31), resulting in twice as many locules compared to wild-type (WT, *SlCLV3*^*FAS*^). The combination of these cis-regulatory alleles in homozygous double mutant plants (*Slclv3*^*fas*^ *Slwus*^*lc*^) produces an enhanced (i.e. synergistic) epistatic effect on locule number that surpasses their combined individual effects (Fig. 1A) (26). Thus, the emergence of *Slclv3*^*fas*^ in the context of the pre-existing *Slwus*^*lc*^ background is thought to have been a key step in the increase in fruit size observed during tomato domestication (31). However, additional cis-regulatory alleles of the *SlCLV3* locus exist (32, 33), and it remains an open question whether this synergistic interaction is specific to *Slclv3*^*fas*^ or whether other cis-regulatory alleles of this gene with varying allelic strengths would also exhibit epistatic enhancement with *Slwus*^*lc*^.

## Epistasis across an allelic series of cis-regulatory mutations

Using natural alleles to investigate the impact of cis-regulatory allelic diversity on epistatic interactions in any system is challenging, due to their varied genetic backgrounds and limited understanding of their phenotypic effects. Previously, we used CRISPR/Cas9 to engineer cis-regulatory deletion mutations that overlapped with the disrupted cis-regulatory sequences of *Slwus*^*lc*^ and *Slclv3*^*fas*^, resulting in mimics of their individual effects in the same genetic background (26, 28). In the same experiment, we engineered an additional 28 *Slclv3* promoter alleles (*Slclv3*^*Pro*^), resulting in a continuum of locule number variation from subtle increases in the proportion of three-locule fruit to strong *Slclv3* null-like effects, shown in fruits that on average contain more than 15 locules (28). Leveraging this genetic resource, and its power to quantify locule number over a wide phenotypic range, we tested whether the *Slwus*^*lc*^ mimic (*Slwus*^*CR-lc*^) consistently enhances the effects of *Slclv3*^*Pro*^ cis-regulatory alleles to the same degree as with *Slclv3*^*fas*^ or whether epistatic interactions are dependent on the allelic strength and/or specific identity of the *Slclv3*^*Pro*^ alleles.

From the pool of available *Slclv3*^*Pro*^ alleles, we selected 12 that represent the full spectrum of locule number variation, including *Slclv3*^*fas*^, and demonstrated that their homozygous mutant effects are reproducible across multiple years and environments (fig. S1A and table S3). This resource allowed us to measure how the magnitude of the epistatic interaction with *Slwus*^*CR-lc*^ changes across this allelic series of cis-regulatory mutants (Fig. 1C). To evaluate the combined effects of *Slclv3*^*Pro*^ and *Slwus*^*CR-lc*^ cis-regulatory alleles, we created all possible double mutant combinations in the same genetic background as the single mutants (Fig. 1B and fig. S1A–B). We then quantified locule numbers from all 2 × 12 = 24 genotypes, including WT and single mutants, across two replicated experiments (Fig. 1B–C and fig. S2A).

We considered several specific hypotheses on how the magnitude of this epistatic interaction (table S2) might change as a function of cis-regulatory allelic strength: the absence of epistasis from *Slwus*^*CR-lc*^ (i.e. additivity), and three modes of epistasis across the *Slclv3*^*Pro*^ allelic series: proportional, constant, and idiosyncratic (Fig. 1D). In proportional epistasis (also known as the multilinear model) (34), the *Slwus*^*CR-lc*^ effect scales linearly with *Slclv3*^*Pro*^ allelic strength, whereas in constant epistasis the *Slwus*^*CR-lc*^ effect is the same for each mutant allele. Idiosyncratic epistasis, on the other hand, is allele-specific in that the *Slwus*^*CR-lc*^ effect varies, potentially in either positive or negative directions, depending on the *Slclv3*^*Pro*^ mutant background (35, 36).

To test these hypotheses, we built a nested family of models and fit them to the log-transformed data using maximum likelihood (Supplementary Materials). This analysis found that although neither the constant nor proportional epistasis models provided a better fit than the additive model (likelihood ratio test, *p*=.88 and *p*=.32, respectively), the additive, constant epistasis, and proportional epistasis models could all be rejected in favor of the idiosyncratic epistasis model (likelihood ratio test, *p*<.0001 against all simpler models). Thus, the effect of *Slwus*^*CR-lc*^ across the *Slclv3*^*Pro*^ allelic series is neither constant nor a simple function of allelic strength but rather varies substantially in an allele-specific manner (Fig. 1E). A notable example is *Slclv3*^*Pro-22*^. While this single mutant displays higher locule numbers than both the *Slclv3*^*fas*^ and *Slclv3*^*fas*^ *Slwus*^*CR-lc*^ genotypes, counter to expectations, in the background of *Slclv3*^*Pro-22*^, *Slwus*^*CR-lc*^ actually decreases locule number, constituting a strong negative idiosyncratic effect (Fig. 1C–E). Moreover, our analysis also shows that the strong positive idiosyncratic effect from *Slwus*^*lc*^ on the *Slclv3*^*fas*^ background was not observed with any other *Slclv3*^*Pro*^ alleles (Fig. 1E). Thus, the combined effect on locule number from *Slclv3*^*fas*^ and *Slwus*^*lc*^ played a unique and critical role in enhancing fruit size during domestication, beyond what their individual effects could achieve.

The idiosyncratic epistasis between *Slwus*^*CR-lc*^ and a subset of specific *Slclv3*^*Pro*^ alleles was surprising given the continuous phenotypic variation produced across the *Slclv3*^*Pro*^ allelic series. This raised the question of whether such unpredictability would be recapitulated with mutations of *SlCLE9,* which enhance the effects of both the *Slclv3* null mutation and the *Slclv3*^*fas*^ cis-regulatory mutation (27). Notably, similar to *Slclv3* null alleles, the expression of *SlCLE9* is upregulated in *Slclv3*^*fas*^ mutant meristems, though to a lesser degree (27). We confirmed this result and further showed that, overall, across the *Slclv3*^*Pro*^ allelic series, *SlCLE9* expression increases as *SlCLV3* expression decreases (fig. S3) as one moves from low to high locule number alleles. These observations suggested that, unlike the idiosyncratic epistasis imposed by *Slwus*^*CR-lc*^ on the *Slclv3*^*Pro*^ allelic series, *Slcle9* could progressively enhance locule numbers across the allelic series, which would support proportional epistasis (Fig. 1D).

**Fig. 2.**
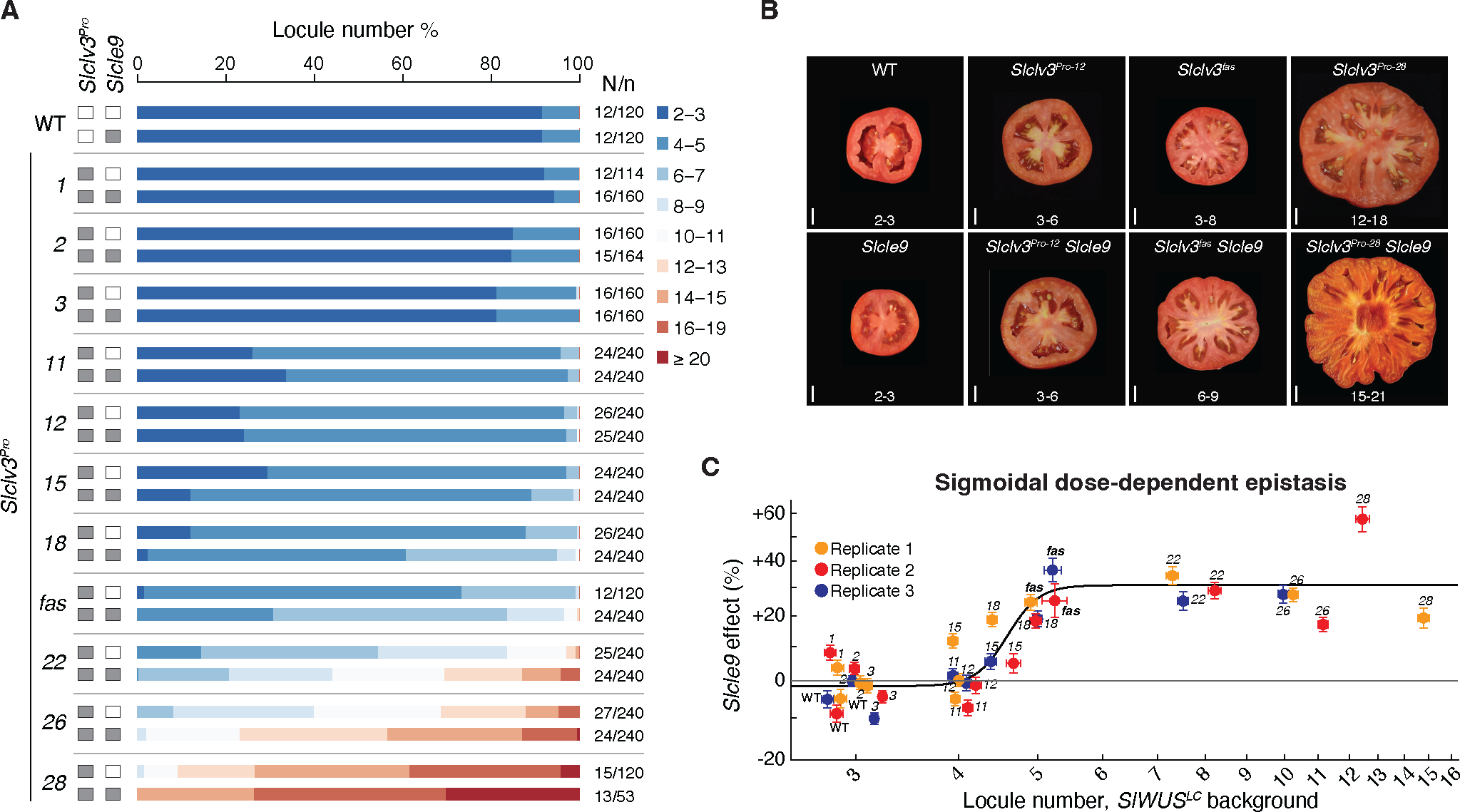
The compensating paralog *SlCLE9* interacts with *SlCLV3* in a sigmoidal dose-dependent epistasis relationship. (**A**) Stacked bar charts show percentage of total fruits for each locule number range of *Slclv3*^*Pro*^ single and *Slclv3*^*Pro*^ *Slcle9* double mutant alleles. White/Gray boxes indicate WT and mutant genotype for each gene, respectively. Number of replicated plants/number fruits (N/*n*). (**B**) Representative fruit images and locule number quantification (mean ±1 standard deviation) showing the effect of *Slcle9* on locule number in WT and the *Slclv3*^*Pro*^ mutants. Scale bars: 1 cm. (**C**) *Slcle9* effect on mean log locule number (*Slcle9 Slclv3*^*Pro*^ double mutants as compared to *SlCLE9 Slclv3*^*Pro*^ single mutants), plotted against the mean log locule number of the corresponding *SlCLE9 Slclv3*^*Pro*^ genetic background (error bars indicate ±1 standard error). Black line indicates the maximum likelihood fit for the sigmoid model. Data are from three replicate trials (see also fig. S2B and tables S2 and S3).

Utilizing the same *Slclv3*^*Pro*^ mutants and approach as for *Slwus*^*CR-lc*^ (Fig. 2A and fig. S1C), we unexpectedly found that for *Slcle9* all of the simpler models were again rejected in favor of the idiosyncratic epistasis model (likelihood ratio test, *p*<.0001 against all simpler models). However, unlike for *Slwus*^*CR-lc*^ where the allele-specific effects varied substantially between phenotypically similar genetic backgrounds, the additive model could be rejected in favor of both the constant and proportional epistasis models (likelihood ratio test, *p*<.0001 for both models), and examination of the estimated epistatic effects between all single and double mutant pairs (table S2) suggested that the *Slcle9* effect varied in a threshold-like manner as a function of *Slclv3*^*Pro*^ allelic strength. In particular, while *Slcle9* had only a minimal effect on locule in the weaker *Slclv3*^*Pro*^ backgrounds (which express *SlCLV3* at near wild-type levels, fig. S3), a larger effect emerged in the stronger, higher locule backgrounds where *SlCLV3* is expressed at a substantially lower level (fig. S3), including *Slclv3*^*fas*^ and the near null mutant *Slclv3*^*28*^ (Fig. 2B). Based on these observations, we fit an additional model where the *Slcle9* effect increases as a sigmoid function of the strength of the *Slclv3*^*Pro*^ background (Fig. 2C). Though the idiosyncratic epistasis model still provided a better fit to the data (likelihood ratio test, *p*<.0001), the sigmoid model provided a better fit than either the constant or proportional epistasis models (likelihood ratio test, *p*<.0001 against both simpler models). Moreover, if we consider the epistatic variance in log locule number as the fraction of the variance that is accounted for by the idiosyncratic epistasis model but not accounted by the additive model, we find that the sigmoid model captures the vast majority of this variance (90.0%, table S2). We thus conclude that while there is a statistically significant idiosyncratic component to the *Slcle9* effect, the overall pattern is a dose-dependent saturating relationship, where the effect of *Slcle9* is negligible until a critical *Slclv3*^*Pro*^ allelic strength (critical degree of *SlCLV3* disruption) is reached. Above this threshold, the effect of *Slcle9* increases and eventually reaches an approximately constant level of enhancement in stronger *Slclv3*^*Pro*^ backgrounds.

## Higher-order mutant combinations reveal additional idiosyncrasy

**Fig. 3.**
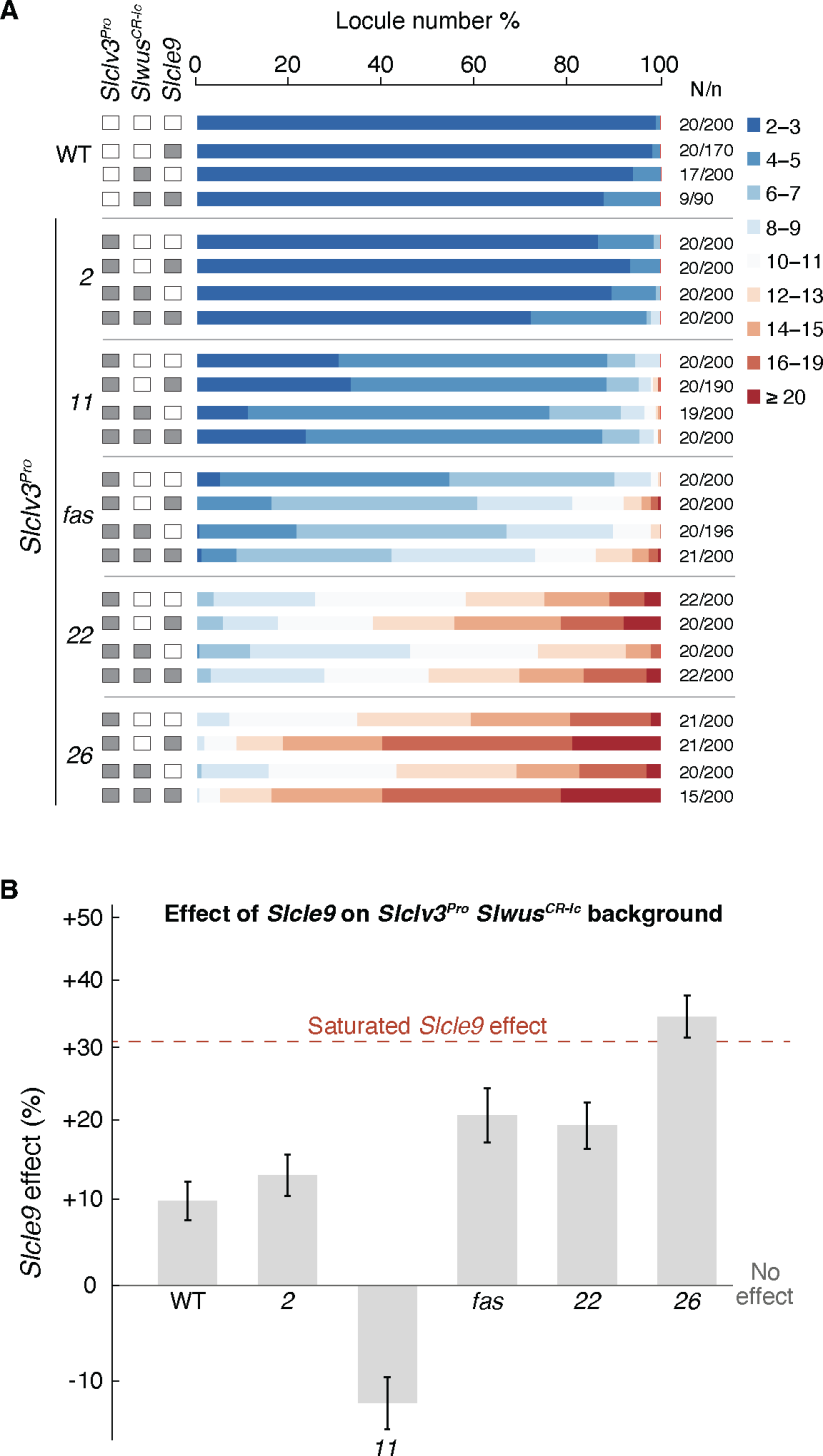
Loss of *SlCLE9* imposes new and unpredicted idiosyncratic effects on *Slclv3*^*Pro*^ *Slwus*^*CR-lc*^ backgrounds. (**A**) Stacked bar charts show percentage of total fruits for each locule number range of WT and all indicated single, double, and triple mutant genotypes. White/Gray boxes indicate WT and mutant genotype for each gene, respectively. Number of replicated plants/fruits (N/*n*). (**B**) *Slcle9* effect on the log mean locule number (*Slcle9 Slwus*^*CR-lc*^ *Slclv3*^*Pro*^ triple mutants as compared to the *SlCLE9 Slwus*^*CR-lc*^ *Slclv3*^*Pro*^ double mutants) in the indicated *SlCLE9 Slwus*^*CR-lc*^ *Slclv3*^*Pro*^ double mutant background (error bars indicate ±1 standard error). Notice the strong negative idiosyncratic epistasis in the *Slclv3*^*Pro-11*^ *Slwus*^*CR-lc*^ background. Black line indicates no effect and the red dashed line indicates the saturated effect of *Slcle9* on *Slclv3*^*Pro*^ based on our previously fit sigmoid model (see also Fig. 2C, fig. S2C and tables S2 and S3).

While our findings show that the effects of *Slcle9* null mutants have a sigmoid epistasis relationship across the *Slclv3*^*Pro*^ allelic series, modern genotypes typically also carry *Slwus*^*lc*^ (31). To evaluate whether this pattern is maintained in the presence of *Slwus*^*CR-lc*^, we constructed and phenotyped a combinatorially complete set of triple mutants using a subset of five mutant *Slclv3*^*Pro*^ alleles with a wide range of allelic strengths (Fig. 3A, 6 × 2 × 2 = 24 total genotypes). Surprisingly, we found new and unpredicted epistatic interactions in these higher-order mutants that were not present in the double mutants. Though the effect of *Slcle9* on locule number is negligible in wild-type, *Slwus*^*CR-lc*^, and weak *Slclv3*^*Pro*^ mutant backgrounds, locule number was enhanced by *Slcle9* in all triple mutants, including with the weak *Slclv3*^*Pro-2*^ allele (Fig. 3A and fig. S2C), and the *Slcle9* effect broadly increased and approached saturation at approximately the level predicted by the sigmoid model (Fig. 3B). Although our previous analyses showed that *Slwus*^*CR-lc*^ had a strong positive and negative idiosyncratic influence on the effects of *Slclv3*^*fas*^ and *Slclv3*^*Pro-22*^, respectively (Fig. 1E), we did not observe strong idiosyncratic epistasis with *Slcle9* and these alleles, even though *Slwus*^*CR-lc*^ was present in the backgrounds of the triple mutants (Fig. 3 and table S2). In contrast, we observed a striking reversal of the *Slcle9* effect on *Slclv3*^*Pro-11*^ in the presence of *Slwus*^*CR-lc*^, where locule number is actually decreased, instead of increased, by the *Slcle9* mutation (Fig. 3B). Consistent with this new idiosyncratic effect, the constant and proportional epistasis models were rejected in favor of the idiosyncratic epistasis model (likelihood ratio test, *p*<.0001 against both simpler models). Taken together, these findings demonstrate that the predictability of epistatic effects and phenotypic outcomes in two-way interactions can be altered in higher-order allelic combinations.

## Discussion

Cryptic mutations, which have subtle or no effect on phenotype (37), are pervasive in genomes, and despite little knowledge about the underlying genes, alleles, and mechanisms, these cryptic background mutations are widely recognized as critical factors that shape the evolutionary trajectories of traits under both natural and artificial selection (2, 38–40). Our observations expose the dynamic role played by epistasis among the natural and cryptic alleles of these genes during tomato domestication. The natural hypomorphic *SlCLE9* allele pre-existed as a cryptic variant in the genome of the wild progenitor of tomato (27, 29), and was followed by *Slwus*^*lc*^, whose subtle influence on locule number likely also persisted cryptically (31). Consequently, the later emergence of *Slclv3*^*fas*^ would have immediately triggered a positive idiosyncratic epistatic interaction with *Slwus*^*lc*^ wherein these new *Slclv3*^*fas*^ mutants displayed a marked increase in locule number that they would not have shown in the absence of these preceding mutations. Thus, the fortuitous *SLCLV3* cis-regulatory allele responsible for the initial and most consequential step in enhancing fruit size by increasing locule number during domestication appears to have had its quantitative effect due to a combination of an unpredictable idiosyncratic interaction with the cryptic gain-of-function *Slwus*^*lc*^ allele as well as alleviation of dose-dependent suppression by the cryptic hypomorphic *SlCLE9*.

The idiosyncratic epistatic effects that we observe here are presumably driven by allele-specific differences in the composition and location of regulatory elements within the *SlCLV3* promoter. However, identifying the causative regulatory elements is difficult both because each mutant allele typically disrupts dozens of transcription factor binding sites (28), and because the regulatory architecture of meristem development remains incompletely understood (24). In light of the remarkable complexity of epistatic interactions arising from a limited number of background mutations and a one-dimensional array of allelic strength, our findings hold ramifications for other organisms and phenotypes, in both natural genetic contexts and genetic engineering. Gene regulatory networks are the foundation of biological systems (41, 42), and these networks depend on intricate signaling and feedback mechanisms, encompassing both positive and negative regulation, between genes and their protein products, often involving paralogs engaged in asymmetrical redundancy relationships (3, 30, 43). Notably, the redundancy relationship between *SlCLV3* and *SlCLE9* is based on a widespread transcriptional compensation mechanism (27, 29, 43, 44), suggesting that similar saturating dose-dependent epistatic interactions are likely to be ubiquitous. However, varying allelic states of redundant paralogs could affect the form of dose-dependent relationships. For example, *SlCLE9* orthologues differ across Solanaceae crops, from the more potent partner of the *SlCLV3* orthologue in groundcherry to the complete loss of this gene in eggplant (29). These varying allelic states are important to consider when designing editing strategies to increase locule number. Likewise, how epistasis is transformed across an allelic series could also be influenced by environmental conditions. We found that the phenotypic effects of both coding and regulatory *SlCLV3* mutations are typically not affected substantially by the environment (28), and although the patterns of epistasis observed in our study might have some dependence on environment, the genotype-specific locule number distributions remained remarkably consistent across different field seasons and locations (fig. S2). Importantly, employing similar methods to those used here provides a path to determine the form of these interactions for other organisms, traits and environments, which would facilitate the fine-tuning of phenotypes in a controlled and quantitative manner.

It is important to acknowledge, however, that the predictability of outcomes when engineering novel alleles and allelic combinations may be influenced by idiosyncratic interactions with other background mutations (2, 45, 46). Indeed, our observation of a new idiosyncratic effect in the *Slclv3*^*Pro*^ *Slwus*^*lc*^ *Slcle9* triple mutants, which was not present in the *Slclv3*^*Pro*^ *Slwus*^*lc*^ double mutants, underscores how predictability of effects from engineered alleles may decay in increasingly divergent genetic backgrounds. A related issue is that natural alleles responsible for phenotypic differences between genotypes and species, which are being increasingly revealed through pan-genomics (32, 33, 47, 48), may be enriched for idiosyncratic effects due to the action of natural or artificial selection (49, 50), as seen with *Slclv3*^*fas*^ and *Slwus*^*lc*^. More broadly, the expected degree of variability in epistatic interactions displayed by different alleles at the same locus, how these epistatic interactions are transformed as a function of allelic strength, and whether these patterns differ between natural versus artificial alleles and regulatory versus coding sequences remain as open questions. While we have shown that our *Slclv3*^*Pro*^ allelic series interacts differently with *Slwus*^*lc*^ (idiosyncratically) versus *Slcle9* (a systematic, dose-dependent response), it will be informative to investigate whether other allelic series will exhibit consistent or distinct patterns of epistatic interaction when the same allelic series is paired with different epistatic partners. Systematic mapping of predictable epistatic interactions, while either minimizing or perhaps leveraging potential idiosyncratic effects, represents a key challenge in current and future endeavors to modify, correct, and optimize traits in agriculture and human health.

## Supplementary Material

Supplementary Material

Table-S1

Table-S4

Table-S3

Table-S2

## Data Availability

Source code for statistical analysis of epistasis models can be found on Zenodo (51). All data are available in the main text or the supplementary materials.
