# Supplementary material for: Idiosyncratic and dose-dependent epistasis drives variation in tomato fruit size
Source: Science. Author manuscript; Available in PMC 2023 Oct 26. (PMC10602613; doi:10.1126/science.adi5222)
Supplement: Supplementary Material [file NIHMS1936328-supplement-Supplementary_Material.pdf]

Science
AAAS

Supplementary Materials for

Idiosyncratic and dose-dependent epistasis drives variation in tomato fruit size

Lyndsey Aguirre[1], Anat Hendelman[2], Samuel F. Hutton[3], David M. McCandlish[2*], Zachary B. Lippman[1,2,4*]

Corresponding authors: mccandlish@cshl.edu, lippman@cshl.edu

The PDF file includes:

Materials and Methods
Figs. S1 to S3
Tables S1 to S4
References 52

Materials and Methods

Plant material and genotyping

Seeds of wild type (*Solanum lycopersicum* cultivar M82, LA3475), *Slwus*$^{CR\text{-}lc}$ (*Solyc02g083950*), *Slcle9* (*Solyc06g074060*), and *Slclv3*, *Slclv3*^{Pro} including *Slclv3*^{fas} (*Solyc11g071380*) alleles in the M82 background were from our own stocks. *Slwus*$^{CR\text{-}lc}$, *Slclv3*, *Slclv3*^{Pro}, *Slclv3*^{fas}, and *Slcle9* mutant alleles were described before (*26-28*).

All *Slclv3*^{Pro} alleles were generated by our CRISPR-Cas9 mutagenesis drive system (26), Briefly, Cas9 positive F1 *Slclv3*^{Pro} plants were grown in the field at Uplands Farm of Cold Spring Harbor Laboratory, New York. F2 progeny plants were genotyped for both *Cas9* and the *SlCLV3* targeted-promoter region to identify non-transgenic, biallelic, and homozygous plants carrying new alleles (primer sequences for genotyping are listed in table S4). Sequencing of new alleles was performed for at least three cloned individuals per putative allele (StrataClone Blunt PCR Cloning Kit, Agilent). Sequences were assembled using Geneious (v.11.1.5) software. To eliminate potential rare CRISPR-Cas9 off-target mutations and to mitigate effects from potential background mutations, homozygous *Slclv3*^{Pro} alleles were isolated from multiple backcrosses to WT and were verified by PCR to no longer contain the *Cas9* transgene. These homozygous *Slclv3*^{Pro} alleles were then used in crosses to both *Slwus*$^{CR\text{-}lc}$ and *Slcle9,* both of which were also generated in the same near isogenic M82 background. *Slclv3*^{Pro} *Slwus*$^{CR\text{-}lc}$ and *Slclv3*^{Pro} *Slcle9* double homozygous individuals were isolated then in the F2 generation, and in later generations, were used in subsequent comparative quantitative locule phenotyping (Fig. S1) as well as in further crosses to generate *Slclv3*^{Pro} *Slwus*$^{CR\text{-}lc}$ *Slcle9* triple homozygous mutants. Due to the phenotypic severity of the *Slclv3*$^{Pro\text{-}29}$ allele, seed stocks for the S*lclv3*$^{Pro\text{-}29}$ *Slwus*$^{CR\text{-}lc}$ *Slcle9* triple mutant were maintained with segregation of the S*lclv3*$^{Pro\text{-}29\ +/-}$ allele in the background of *Slwus*$^{CR\text{-}lc}$ *Slcle9*, and triple homozygous mutant plants for this allele were isolated genotypically in each generation and were verified phenotypically by the severity of both vegetative and fruit fasciation.

Growth conditions and phenotyping

Seeds were either germinated on moistened filter paper at 28 °C in the dark and later transferred to soil at three days post germination (dpg) or directly sown in soil in 96-cell plastic flats and grown to 4~5-week-old seedlings in the greenhouse. Seedlings were then transplanted to 4L pots in the greenhouse for crossing purposes or directly to the fields at Cold Spring Harbor Laboratory, New York or at The University of Florida Gulf Coast Research and Education Center. The greenhouse condition is long-day (16 h light, 26-28 °C / 8 h dark, 18-20 °C; 40-60% relative humidity) with natural light supplemented with artificial light from high-pressure sodium bulbs (~250 µmol m^{-2} s^{-1}). Plants in the fields were grown under drip irrigation and standard fertilizer regimes, and were used for quantifications of fruit locule number. To quantify fruit locule number, fruits were harvested from multiple inflorescences and dissected horizontally to allow quantification of locule number. For each genotype, locules were counted from ~100-300 fruits from ~15-30 individual plants on average (table S3). For the *Slclv3*$^{Pro\text{-}29}$ *Slcle9* double mutants and the *Slclv3*$^{Pro\text{-}29}$ *Slwus*$^{CR\text{-}lc}$ *Slcle9* triple mutants, fruit set was low due to fertility defects from severe fasciation. Fruit locule counts for these genotypes were either aggregated from all seasons and treated as a single replicate (fig. S2B) or carpel numbers were counted from flowers to supplement for low locule count data (fig. S2C). To quantify ovary carpel number, ovaries from multiple inflorescences were dissected under a conventional stereoscope and carpel number was quantified separately from ~10 plants per genotype. As we previously observed that environmental conditions can have minor effects on epistatic relationship among *Slclv3* promoter mutant alleles (28), to

ensure the robustness of our results to environmental variation, for each of the replicated experiments, the plants were grown in two locations (New York and Florida, representing different soil conditions) and over multiple years and growing seasons. For the *Slclv3^{Pro} Slwus$^{CR\text{-}lc}$* experiments, the locule number phenotyping assays were repeated over two independent field seasons: once at Cold Spring Harbor Laboratory's fields in the summer season (2021) and once at The University of Florida-Gulf Coast Research and Education Center fields in the fall season (2021). Locule number assays for the *Slclv3^{Pro} Slcle9* experiments were performed once at Cold Spring Harbor Laboratory's fields in the summer season (2020) and twice at The University of Florida-Gulf Coast Research and Education Center fields in the spring season (2020 and 2021). Locule number assays for the *Slclv3^{Pro} Slwus$^{CR\text{-}lc}$ Slcle9* experiment were also performed at The University of Florida-Gulf Coast Research and Education Center fields in the spring season (2022).

RNA extraction and Quantitative RT-PCR (qPCR)

For gene expression analysis, seeds were germinated on moistened filter paper on Petri dishes at 28 °C in dark. At three dpg, seedlings at a similar developmental stage were transferred to soil in 96-cell plastic flats and grown in the greenhouse. Shoot apices, at the floral meristem developmental stage (meristem maturation staging determined according to (52)), including the first floral meristem and both vegetative and inflorescence sympodial meristems, were collected under a stereoscope and immediately flash-frozen in liquid nitrogen. Seven to ten apices were combined as one biological replicate, and two or three replicates were collected for each genotype. Total RNA was extracted using TRIzol® Reagent (Invitrogen) and 400 ng of total RNA was used for cDNA synthesis using the SuperScript IV VILO Master Mix (Invitrogen). qPCR was performed with gene-specific primers using the Fast SYBR Green Master Mix (Applied Biosystems) reaction system on the QuantStudio 6 Real-Time system (Applied Biosystems). *SlUbiquitin* (*Solyc01g068045*) gene was used as the internal control (all primers used in this study are listed in table S4).

Modeling of epistasis

Because the effects of mutations on locule number interact approximately multiplicatively and the locule number distribution within each line is approximately log-normally distributed, we analyzed the genetic architecture of locule number by conducting least-squares fits on log locule number. More precisely, for each fruit *i* we modeled locule number *yi* as

$$\log(y_i) = \beta_{wt} + \beta_{lc}x_{i,lc} + \beta_{cle9}x_{i,cle9} + \sum_j \beta_j x_{i,j} + \sum_j e_{lc,j}\, x_{i,lc}x_{i,j} + \sum_j e_{cle9,j}x_{i,cle9}x_{i,j} + e_{lc,cle9}x_{i,lc}x_{i,cle9} + \sum_j e_{lc,cle9,j}\, x_{i,lc}x_{i,\mathrm{cle9}}x_{i,j} + \epsilon_i$$

where β_{wt} controls the wild type locule number, β_{lc} controls the effect of the *Slwus$^{CR\text{-}lc}$* allele, β_{cle9} controls the effect of the *Slcle9* allele, β_j controls the effect of the j-th mutant *Slclv3^{Pro}* allele, $e_{lc,j}$ controls the pairwise interaction between *Slwus$^{CR\text{-}lc}$* and the j-th mutant *Slclv3^{Pro}* allele, $e_{cle9,j}$ controls the pairwise interaction between *Slcle9* and the j-th mutant *Slclv3^{Pro}* allele, $e_{lc,cle9}$ controls the pairwise interaction between *Slwus$^{CR\text{-}lc}$* and *Slcle9*, and $e_{lc,cle9,j}$ controls the three-way interaction between *Slwus$^{CR\text{-}lc}$*, *Slcle9*, and the j-th mutant *Slclv3^{Pro}* allele. In the above, the $x_{i,k}$ are indicator variables that take the value 1 if fruit *i* carries allele *k*, and 0 otherwise, and ϵ_i is normally distributed and independent between fruit, with mean 0 and variance σ^2 (so that the maximum likelihood fit of the above model also yields the least squares solution). For the *Slwus$^{CR\text{-}lc}$* and

Slcle9 experiments, data was pooled across both of the replicates for *Slwus*$^{CR\text{-}lc}$ and all three replicates for *Slcle9*.

While this general model allows a different pairwise and three-way interaction effect for each *Slclv3*^{Pro} allele, we also considered less complex models by constraining the values of the interaction coefficients. Specifically, the non-epistatic model sets the value of all interaction terms $e_{lc,j}$, $e_{cle9,j}$, $e_{lc,cle9}$, and $e_{lc,\, cle9,\, j}$ to 0; the constant epistasis model sets e.g. all the $e_{lc,\, j}$ to the same value; and the proportional epistasis model sets e.g. $e_{lc,\, j} = m\, \beta_j$, where m is a free parameter, so that the magnitude of the epistatic interaction is proportional to the strength of the genetic background. Noting that the proportional and constant epistasis models are nested within the saturated (idiosyncratic epistasis) model and the non-epistatic model is nested within the proportional and constant epistasis models, we compared these models using likelihood ratio tests. For the analysis of the interaction between *Slcle9* and the *Slclv3*^{Pro} alleles, we also considered a model with a saturating interaction term of the form $e_{cle9,j} = a\frac{1}{1+e^{-m\,\beta_j+b}} + c$, which is nested between the constant epistasis and idiosyncratic epistasis models and can arbitrarily closely approximate the proportional epistasis model. For the triple-mutant data we are specifically interested in the effect of *Slcle9* and the interaction between *Slcle9* and the various *Slclv3*^{Pro} mutants when these mutations occur on a *Slwus*$^{CR\text{-}lc}$ background. For the triple mutants, we thus consider only the genotypes containing *Slwus*$^{CR\text{-}lc}$ and fix all of β_{wt}, β_{lc}, β_{cle9} and the β_j to be zero. Then the triple-mutant idiosyncratic epistasis model contains all the remaining terms as free parameters, the proportional epistasis model fixes $e_{lc,\, cle9,\, j} = m\, e_{lc,\, j}$, the constant epistasis model sets all the $e_{lc,\, cle9,\, j}$ equal to the same value, and the no epistasis model sets all the $e_{lc,\, cle9,\, j}$ equal to zero.

In order to provide an additional metric by which to compare these models, we note that the idiosyncratic epistasis model is a fully saturated model with one parameter per genotype and thus captures all possible forms of epistasis, whereas the no epistasis (additive) model does not contain any epistasis. We can therefore quantify the variance in log locule number due to epistasis as the difference between the mean squared error of the idiosyncratic model and the mean squared error of the additive model. The improvement of the mean squared error of any other model relative to the additive model can then be compared to this total epistatic variance to compute the fraction of epistatic variance captured by that model. These values and other metrics of model performance (AICc, BIC) are included in table S2.

Finally, while our main analysis addresses the pattern of epistasis across the *Slclv3*^{Pro} allelic series as a whole, it also may be of interest to ask whether each *Slclv3*^{Pro} mutation has a detectable pairwise or three-way interaction with *Slwus*$^{CR\text{-}lc}$ or *Slcle9* when considered separately from the other *Slclv3*^{Pro} mutations. To answer this question, for each of the three experiments and for each mutant *Slclv3*^{Pro} allele, we fit an additional series of models that included only that particular mutant *Slclv3*^{Pro} allele (table S2). More precisely, for each experiment we fit a series of models of the form:

$$\log(y_i) = \beta_0 + \beta_{lc}x_{i,lc} + \beta_{cle9}x_{i,cle9} + \beta_j x_{i,j} + e_{lc,j}x_{i,lc}x_{i,j} + e_{cle9,j}x_{i,cle9}x_{i,j} + e_{lc,cle9}x_{i,lc}x_{i,cle9} + e_{lc,cle9,j}x_{i,lc}x_{i,\mathrm{cle9}}x_{i,j} + \epsilon_i$$

where the y_i consist of all the data from that experiment that included no mutant *Slclv3*^{Pro} alleles other than the allele of interest j. Here, the $x_{i,k}$ take the value -1 if allele *k* is wildtype in fruit *i* and +1 if it is mutant, and β_0 gives the inferred mean phenotype across the four genotypes involved in

a specific pairwise interaction or the 8 genotypes involved in a specific three-way interaction. The additive effect at each locus was then determined by fitting the model with all of the interaction terms set to zero, and the significance of the additive effect was determined via a likelihood ratio test against the model with the corresponding β_k set to zero. Pairwise interaction coefficients were likewise determined by fitting a model where the three-way interaction coefficient $e_{lc,\, cle9,\, j}$ was set to zero and the statistical significance of each pairwise interaction was determined via a likelihood ratio test against a model fit with the corresponding double mutant interaction term set to zero. For the triple-mutant experiment, the significance of the three-way interaction coefficient was determined by a likelihood ratio test against the model with $e_{lc,\, cle9,\, j}$ set to zero. Note that because in this analysis the main effects β_{lc}, and β_{cle9} are determined separately for each of the *Slclv3*^{Pro} mutant alleles, changes in the mutational effects of *Slwus*$^{CR\text{-}lc}$ and *Slcle9* across the allelic series typically appear both as apparent changes in these main effects across the *Slclv3*^{Pro} mutations as well as more explicitly as changes in the magnitude of the estimated interaction terms.

Supplementary Figure 1

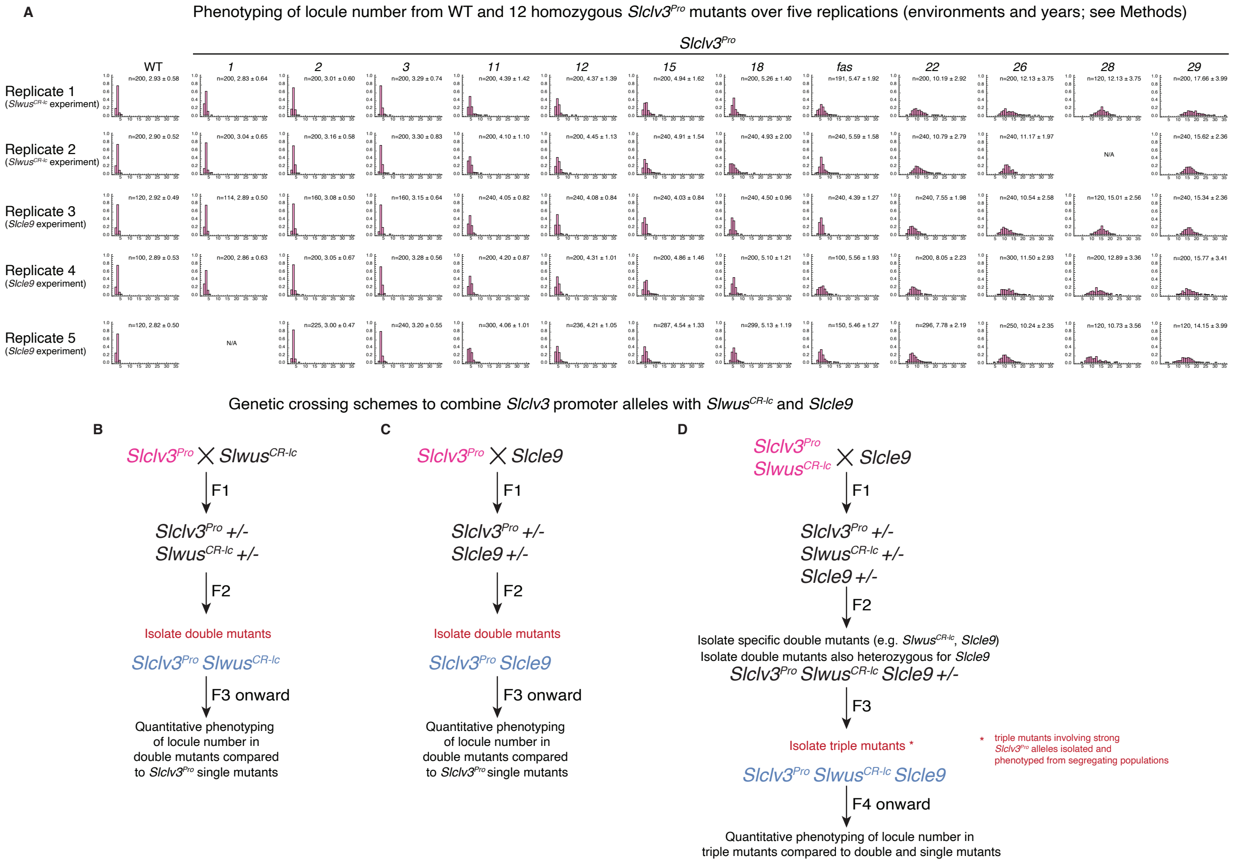

Fig. S1. Genetic schemes used to generate double and triple mutant allele combinations for phenotypic analysis, and reproducibility of phenotypic effects from the *Slclv3^{Pro}* allelic series. (**A**) Histograms from five replicated trials showing the distribution of locule numbers for WT and each homozygous *Slclv3^{Pro}* mutant genotype from different years and environments (Replicate 1- New York, summer 2021; Replicate 2- Florida, fall 2021; Replicate 3- Florida, spring 2021; Replicate 4- New York, summer 2020; Replicate 5- Florida, spring 2020; Supplementary Materials, Growth conditions and phenotyping; table S3). Data demonstrates the consistency of phenotypic effects from each *Slclv3^{Pro}* mutant allele, as well as reproducibility of the inter-allelic relationships. N/A indicates absence of dataset. Top right shows number of fruits (*n*) and mean ±1 standard deviation for each genotype. (**B-D**) Genetic crossing scheme for generating *Slclv3^{Pro} Slwus^{CR-lc}* and *Slclv3^{Pro} Slcle9* double mutant genotypes (B, C respectively), and *Slclv3^{Pro} Slwus^{CR-lc} Slcle9* triple mutant genotypes (D). All mutant alleles were Cas9 negative and backcrossed at least twice to WT before crossing to other mutant backgrounds (Supplementary Materials, Plant material and genotyping).

Supplementary Figure 2

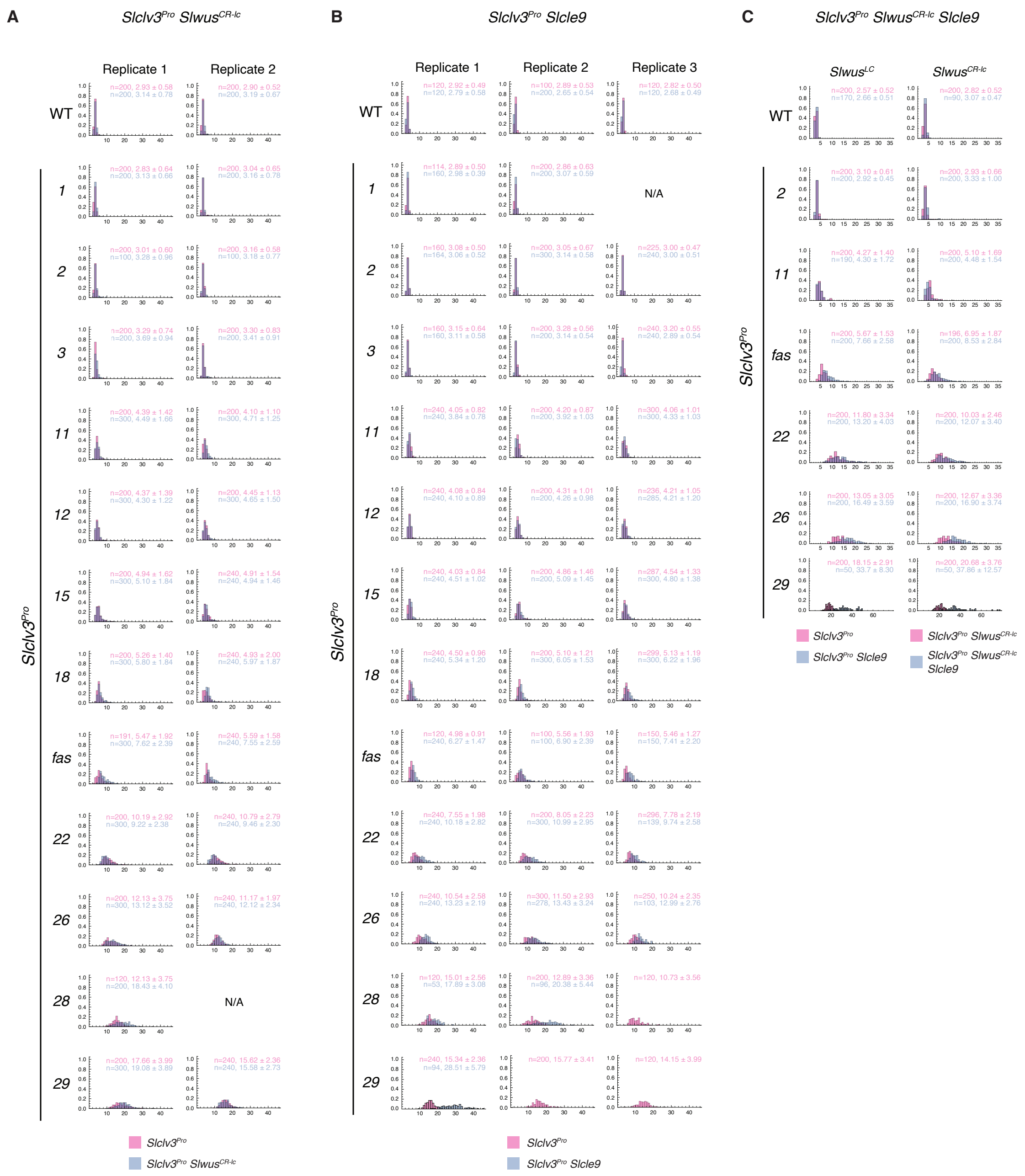

Fig. S2. Effects of *Slwus*$^{CR\text{-}lc}$ and *Slcle9* on fruit locule number across the *Slclv3*^{Pro} allelic series. (**A**) Histograms of the distribution of locule number for WT and the indicated *Slclv3*^{Pro} single mutants (pink bars) and corresponding *Slclv3*^{Pro} *Slwus*$^{CR\text{-}lc}$ double mutants (light blue bars) in two replicated trials (Replicate 1- New York, summer 2021; Replicate 2- Florida, fall 2021; Supplementary Materials, Growth conditions and phenotyping; table S3). The *Slclv3*$^{Pro\text{-}29}$ allele is a 7.3kb deletion that removes entirety *SlCLV3* coding sequence and its *cis*-regulatory regions. This null allele, has the same effect on locule number as CRISPR-Cas9 generated *Slclv3* coding mutations that cause a frame-shift. Top right indicates number of fruits (*n*) and mean ±1 standard deviation for each genotype. (**B**) Histograms of the distribution of fruit locule number for WT, the indicated *Slclv3*^{Pro} single mutants (pink bars), and the corresponding *Slclv3*^{Pro} *Slcle9* double mutants (light blue bars) in three replicated trials (Replicate 1- Florida, spring 2021; Replicate 2- New York, summer 2020; Replicate 3- Florida, spring 2020; Supplementary Materials, Growth conditions and phenotyping; table S3). Top right indicates number of fruits (*n*) and mean ± 1 standard deviation for each genotype. The *Slclv3*$^{Pro\text{-}29}$ *Slcle9* data presented in Replicate 1 is an aggregation from fruits harvested across multiple field seasons (table S3). (**C**) Histograms of the normalized distributions of fruit locule number of *Slclv3*^{Pro} *Slwus*$^{CR\text{-}lc}$ double mutants (pink bars) and *Slclv3*^{Pro} *Slwus*$^{CR\text{-}lc}$ *Slcle9* triple mutants (light blue bars). Top right indicates number of fruits (*n*) and mean ±1 standard deviation for each genotype. The *Slclv3*$^{Pro\text{-}29}$ null mutant alone and in combination with *Slwus*$^{CR\text{-}lc}$ and *Slcle9* as double mutant sets fewer fruits from flowers compared to other *Slclv3*^{Pro} mutants. Thus, carpel numbers from fully developed flowers were quantified as a supplement to locule numbers (Florida, spring 2022; Supplementary Materials, Growth conditions and phenotyping; table S3).

Supplementatry Figure 3

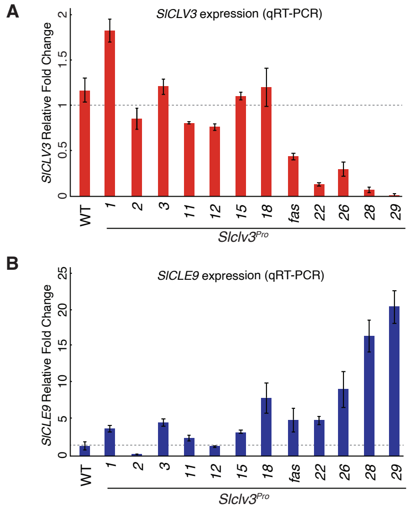

Fig. S3. Expression analysis of *SlCLV3* and *SlCLE9* in reproductive meristem tissue across homozygous mutants of the *Slclv3^{Pro}* allelic series. (**A-B**) qRT-PCR showing expression of *SlCLV3* (A) and *SlCLE9* (B) in WT and the indicated *Slclv3^{Pro}* mutant genotypes. Values are means ± 1 standard error from three biological replicates of pooled reproductive meristems (Supplementary Materials, RNA extraction and Quantitative RT-PCR (qPCR); table S3). Expression is normalized to the control gene *SlUBIQUITIN* and shown as fold change relative to WT.

Captions for Supplementary data

Table S1.
Allelic information for all single, double, and triple mutants used in this study.

Table S2.
Model comparison and supplemental statistical analysis of pairwise and three-way epistasis.

Table S3.
Locule number counts raw data from all seasons and qRT results.

Table S4.
Oligonucleotide primers used in this study.
